# Artificial Intelligence and Machine Learning Technology Driven Modern Drug Discovery and Development

**DOI:** 10.3390/ijms24032026

**Published:** 2023-01-19

**Authors:** Chayna Sarkar, Biswadeep Das, Vikram Singh Rawat, Julie Birdie Wahlang, Arvind Nongpiur, Iadarilang Tiewsoh, Nari M. Lyngdoh, Debasmita Das, Manjunath Bidarolli, Hannah Theresa Sony

**Affiliations:** 1 Department of Pharmacology, North Eastern Indira Gandhi Regional Institute of Health and Medical Sciences (NEIGRIHMS), Mawdiangdiang, Shillong 793018, Meghalaya, India; 2 Department of Pharmacology, All India Institute of Medical Sciences (AIIMS), Virbhadra Road, Rishikesh 249203, Uttarakhand, India; 3 Department of Psychiatry, All India Institute of Medical Sciences (AIIMS), Virbhadra Road, Rishikesh 249203, Uttarakhand, India; 4 Department of Psychiatry, North Eastern Indira Gandhi Regional Institute of Health and Medical Sciences (NEIGRIHMS), Mawdiangdiang, Shillong 793018, Meghalaya, India; 5 Department of Medicine, North Eastern Indira Gandhi Regional Institute of Health and Medical Sciences (NEIGRIHMS), Mawdiangdiang, Shillong 793018, Meghalaya, India; 6 Department of Anesthesiology, North Eastern Indira Gandhi Regional Institute of Health and Medical Sciences (NEIGRIHMS), Mawdiangdiang, Shillong 793018, Meghalaya, India; 7 Department of Computer Science and Engineering, Vellore Institute of Technology, Vellore Campus, Tiruvalam Road, Katpadi, Vellore 632014, Tamil Nadu, India

**Keywords:** artificial intelligence, machine learning, drug discovery, virtual screening, QSAR, QSPR, algorithms, neural networks

## Abstract

The discovery and advances of medicines may be considered as the ultimate relevant translational science effort that adds to human invulnerability and happiness. But advancing a fresh medication is a quite convoluted, costly, and protracted operation, normally costing USD ~2.6 billion and consuming a mean time span of 12 years. Methods to cut back expenditure and hasten new drug discovery have prompted an arduous and compelling brainstorming exercise in the pharmaceutical industry. The engagement of Artificial Intelligence (AI), including the deep-learning (DL) component in particular, has been facilitated by the employment of classified big data, in concert with strikingly reinforced computing prowess and cloud storage, across all fields. AI has energized computer-facilitated drug discovery. An unrestricted espousing of machine learning (ML), especially DL, in many scientific specialties, and the technological refinements in computing hardware and software, in concert with various aspects of the problem, sustain this progress. ML algorithms have been extensively engaged for computer-facilitated drug discovery. DL methods, such as artificial neural networks (ANNs) comprising multiple buried processing layers, have of late seen a resurgence due to their capability to power automatic attribute elicitations from the input data, coupled with their ability to obtain nonlinear input-output pertinencies. Such features of DL methods augment classical ML techniques which bank on human-contrived molecular descriptors. A major part of the early reluctance concerning utility of AI in pharmaceutical discovery has begun to melt, thereby advancing medicinal chemistry. AI, along with modern experimental technical knowledge, is anticipated to invigorate the quest for new and improved pharmaceuticals in an expeditious, economical, and increasingly compelling manner. DL-facilitated methods have just initiated kickstarting for some integral issues in drug discovery. Many technological advances, such as “message-passing paradigms”, “spatial-symmetry-preserving networks”, “hybrid de novo design”, and other ingenious ML exemplars, will definitely come to be pervasively widespread and help dissect many of the biggest, and most intriguing inquiries. Open data allocation and model augmentation will exert a decisive hold during the progress of drug discovery employing AI. This review will address the impending utilizations of AI to refine and bolster the drug discovery operation.

## 1. Introduction

**Figure 1:**
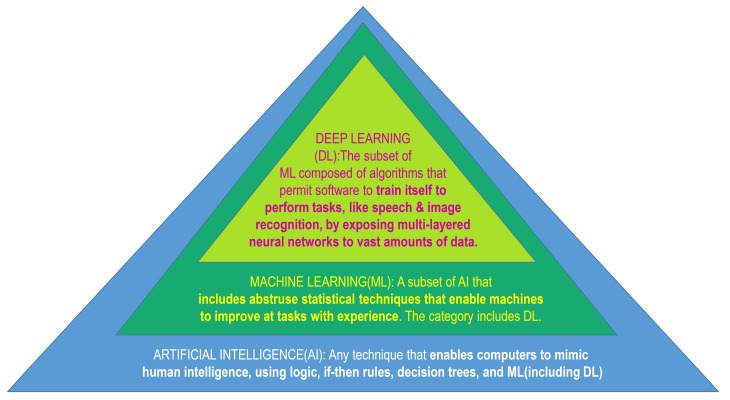
Conceptual Interrelationships between Artificial Intelligence(AI), Machine Learning(ML), & Deep Learning(DL) for drug development.

**Figure 2:**
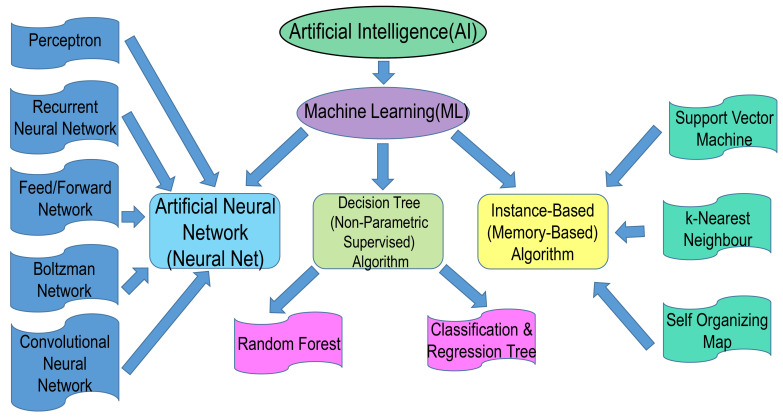
A Summarized Notion of AI & ML Tools engaged in Drug Discovery & Development.

**Figure 3:**
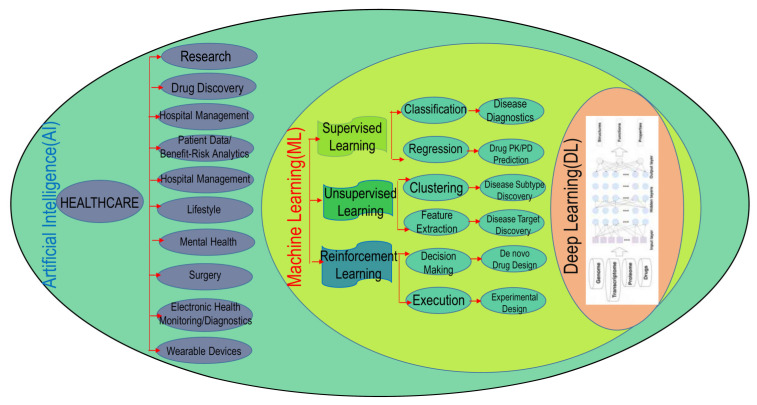
Links between AI, ML & DL for drug development.

The course of research and development of drugs is comprised of drug target recognition, target authentication, hit-to-lead fructification, lead refinement, preclinical molecule determination, and preclinical evaluation, as well as clinical testing. To advance a new prescription drug to market, the mean pretax spending is almost USD 2.6 billion [1], requiring roughly 10–15 years [2]. However, considering the huge financial stakes, the predicted clinical approval realization frequency for novel small agents during the course of discovery and development of drugs is a meagre 13%, with a rather steep possibility of ultimate non-fruition. The advance of computer-enabled drug design technology has been hailed as the most resourceful method for altering this bleak scenario dependent upon prudent navigation in the development process [3]. The methodology pertinent to drug discovery as well as the associated computer-enabled drug design approaches can be located in the treatise “Computer-Assisted Drug Design” [4]. The computational approaches assure a methodical appraisal of the molecular attributes (such as physicochemical properties, selectivity, side effects, bioactivity, and pharmacokinetic parameters) at the speculative level, in concert with engendering optimized molecules having agreeable attributes in silico. Moreover, computational approaches with multi-objective refinement can be engaged to reduce the failure frequency of the preclinical lead molecules. In the vista of drug design, artificial intelligence (AI) invokes the use of computer software programs that evaluate, learn, and reveal pharmaceutical-associated big data to unravel new medicine molecules, by assimilating the advances in machine learning (ML) in a highly unified and mechanized way [5]. Stemming out from the advancement of ML schemes and the growth of chemical and pharmacological information, the AI paradigms have carved out a niche in the arena of drug design for a data-impelled computational process. In comparison with conventional approaches, ML-facilitated approaches, as an offshoot of AI, do not bank upon the theoretical progress of the convoluted and established physico-chemical tenets, but apportion greater emphasis on the metamorphosis of colossal biomedical big data into new enlightenment and sustainable expertise (Figure 1). Typical algorithms synonymous with ML include: Logistic Regression (LR), Naive Bayesian Classification (NBC), k Nearest Neighbor (KNN), Multiple Linear Regression (MLR), Support Vector Machine (SVM), Probabilistic Neural Network (PNN), Binary Kernel Discrimination (BKD), Linear Discriminant Analysis (LDA), Random Forest (RF), Artificial Neural Network (ANN), Partial Least-Squares (PLS), Principal Component Analysis (PCA), and the like [6,7]. In current times, AI technologies, specifically the Deep Learning (DL) paradigms, exhibit tremendous promise in designing drugs, due to their impressive generalization and feature extrication power. Conventional ML approaches employ manually crafted attributes, while the DL approaches can learn features from the input information in an automated fashion, leading to reorganization of simple attributes into convoluted characteristics via multi-layer attribute extrication (Figure 1). Moreover, the DL approaches commonly exhibit less generalization errors than the conventional ML techniques, which facilitates them in obtaining more beneficial outputs on some criterion or competitive assessments. As an instance, George Dahl’s team claimed the Merck Molecular Activity Challenge through implementing the AI technology, specifically the DL algorithms [8]. Owing to the aforementioned conveniences, the DL technique as a data mining approach has demonstrated huge prospects in the drug-designing arena. The DL paradigms are generally comprised of Deep Neural Networks (DNN), Convolutional Neural Networks (CNN), Neural Networks (RNN), autoencoders, and Restricted Boltzmann Machines (RBN). A brisk critique of DL algorithms can be located somewhere else [9,10,11], with a more comprehensive preface to DL methodologies in the treatise “Deep Learning” [12]. This review acquaints the reader with the AI models related to the arena of drug design, and provides an exclusive spotlight on the implementation of DL algorithms in new drug discovery and development. The drug discovery, drug design topics and AI approaches are summarized in Figure 2 and Figure 3.

Also, this review familiarizes ML blueprints linked to drug design schemes, including approaches for the molecular depiction, transfer learning for low data, cross-validation strategy and dexterity of training the deep neural networks (DNNs). Lastly, this review encapsulates the utilities of AI in the arena of drug design and provides a futuristic panorama of the outlook of AI in drug discovery and advancement.

### 1.1. Artificial Intelligence: Facts to Ponder

The last several years have witnessed a radical data digitalization escalation in the pharmaceutical arena. But such digitalization has arrived in response to the demand of amassing, investigating, and engaging that expertise to dissect convoluted clinical issues [13]. This encourages the utilization of AI, as it can process huge quantities of data with augmented mechanization [14]. AI follows a technology-enabled approach invoking multiple cutting-edge tools and networks simulating human intelligence. At the same time, it does not prompt fears of superseding human physical existence, altogether [15,16]. AI exploits software and systems which are enabled to decipher and then trained by input data to arrive at autonomous outcomes for realizing definite aims. As pointed out in this review, its utilization has seen a progressive augmentation in the pharmaceutical sector. As per the McKinsey Global Institute, the swift progression in AI-directed mechanization will probably be thoroughly altering the societal work culture [17,18].

### 1.2. AI: Networks and Tools

AI engages various method domains, like knowledge depiction, solution exploration, deduction, and amidst them, is itself an axiomatic exemplar of ML. ML utilizes algorithmic logic capable of detecting trends in a cluster of data which can be further categorized. An entity of ML is deep learning (DL), which invokes artificial neural networks (ANNs). These networks involve a group of intercommunicating refined computing components engaging ‘perceptrons’, comparable to neurons in human nervous tissue, and simulating the transmittal of electrical excitations within the human CNS [19]. ANNs comprise a group of nodes, with individual nodes accepting a distinct input, and finally transforming inputs to output, either singly or multilinked, utilizing algorithms to decode problems [20]. ANNs comprise many varieties, like multilayer perceptron (MLP) networks, recurrent neural networks (RNNs), and convolutional neural networks (CNNs), engaging either supervised or unsupervised training operations [21,22]. Such MLP networks have utilities inclusive of pattern detection, optimization facilities, process determination, and controls that are generally trained using supervised training algorithms functioning unidirectionally, and may be utilized as universal pattern classifiers (UPCs) [23]. RNNs are network systems possessing a closed loop, having the power to cram and hoard data, like Boltzmann constants as well as Hopfield networks [23,24]. CNNs are an array of dynamic mechanisms engaging local connections, each determined by its topological architecture, with utilities in image and video signal refinement, biological system simulation, handling complex central neuronal activities, pattern appreciation, and refined signal processing [25]. The highly convoluted schemes comprise Kohonen networks, Radial Basis Function (RBF) networks, Learning Vector Quantization (LVQ) networks, counter-propagation networks (CPNs), and Adaptive Linear Neuron or later Adaptive Linear Element (ADALINE) networks [21,23]. Instances of AI-based method domains are depicted in Figure 2. Various algorithms have been crafted depending upon the interconnections that constitute the fundamental framework of AI paradigms. An instance of this advanced tool employing AI scheme is the Watson supercomputer by International Business Machines (IBM) (IBM, New York, NY, USA). This computing infrastructure was devised to support the scrutiny of a patient’s clinical data and its interrelationships against a mammoth database, culminating in determining intervention modalities for cancer. Such a facility may in addition be also utilized for the swift revelation of afflictions. Its utility was established by its power to identify breast cancer within 60 s [26,27].

## 2. Futuristic Applications of AI in Drug Design

Drug designing and development is an important area of research for pharmaceutical companies and chemical scientists. In order for a molecule to have any potential as a drug target it must be “druggable”. In the post-genomic era, drug discovery has shifted towards applying new design principles to molecules or new strategies to bind, modulate, or degrade challenging biological targets for future innovative medicines. Traditionally, the pharmaceutical industry has been focusing on developing orally bioavailable small molecules with established targets (druggable targets). Based on the physicochemical profiles of Phase II drugs, Lipinski’s Rule of Five (Ro5) was developed in 1997. Ro5 predicts that poor absorption or permeation is more likely when there are more than five hydrogen-bond donors (HBD > 5), more than ten hydrogen-bond acceptors (HBA > 10), the molecular weight is greater than 500 Da (MW > 500), and the calculated Log P is greater than five (cLogP > 5). Since then, Ro5 has served as a guide for designing developable molecules during drug discovery. While the efforts to discover small molecule Ro5 compounds that interact with established “druggable” targets have been productive, there is an increased demand for innovation to engage newer targets for transformative medicines. As a result, identification and validation of novel biological targets have become a key focus in the early stages of drug discovery. Molecular modalities beyond bRo5, small molecules via nontraditional modes of action (e.g., protein–protein interaction or PPI modulators) include bifunctional bRo5 small molecules (e.g., protein-targeted chimeras or PROTACs), peptides/peptidomimetics, and oligonucleotides (ONs). Carbohydrate-based drug discovery is an up-and-coming area of research in medicinal chemistry. Bioactive carbohydrates have opened up a new source for drug development. More than 170 carbohydrate-based drugs have been successfully approved as anticoagulants, antitumor agents, antidiabetic agents, antibiotics, antiviral agents, and vaccines. However, most carbohydrates have low druggability. New methods and strategies to improve carbohydrates’ druggability are in high demand. Lipids are essential for life. They store energy, constitute cellular membranes, serve as signaling molecules, and modify proteins. In the long history of lipid research, many drugs targeting lipid receptors and enzymes that are responsible for lipid metabolism and function have been developed and applied to a variety of diseases. Lipid signaling pathways (prostanoids, leukotrienes, epoxy fatty acids, sphingolipids, lysophospholipids, endocannabinoids, and phosphoinositides) and lipid signaling proteins (lysophospholipid acyltransferases, phosphoinositide 3-kinase, and G protein-coupled receptors (GPCRs) offer a wide array of druggable targets. However, the vast majority of the targets of approved drugs are proteins. A druggable protein is one that possesses folds that favor interactions with small drug-like molecules, be they endogenous or extraneous, and therefore is one that contains a binding site. These binding sites are expected to have certain attributes that enable high affinity site-specific binding with the drug-like molecule. As with all drug targets, a potential protein drug target must be linked to a disease process. Currently, there is a lack of knowledge about both the number of proteins that modern pharmaceuticals act upon and the number of potentially druggable proteins. The development of efficient and advanced systems for the targeted delivery of therapeutic agents with maximum efficiency and minimum risks has imposed a great challenge upon chemical and biological scientists. Researchers across the globe engage in traditional computational approaches like virtual screening (VS) and molecular docking to identify and characterize protein–protein as well as drug–protein interactions. But these approaches are imprecise and inaccurate. In addition, complex and big data from genomics, proteomics, microarray data, and clinical trials also impose an impediment in the drug discovery pipeline. AI and ML technology are innovative approaches that play a crucial role in drug discovery and development. This section of the review article provides crucial insights about how artificial neural networks (ANNs) and deep learning (DL) algorithms have modernized the techniques of elucidating the structure and function of proteins, unravelling hits, hit-to-lead optimization algorithmic schemes, and in silico evaluation of ADME/T properties. ML and DL algorithms have been implemented in several drug discovery processes such as peptide synthesis, structure-based virtual screening, ligand-based virtual screening, toxicity prediction, drug monitoring and release, pharmacophore modelling, quantitative structure–activity relationship (QSAR), drug repositioning, poly-pharmacology, and physico-chemical activity. Evidence from the past strengthens the implementation of AI and DL in this field. Moreover, novel data mining, curation, and management techniques provide critical support to recently developed modelling algorithms. In summary, AI and DL advancements provide an excellent opportunity for a rational drug design and discovery process, one which will eventually impact mankind.

### 2.1. The Structure and Function of Proteins

#### 2.1.1. Prognostication of Protein Folding from Sequence (Predicting the 3D Structure of a Target Protein)

Most diseases are linked to dysfunctional proteins. By scrutinizing protein architectures, the structure-based drug design blueprints can be employed to originate the active small compounds for the protein targets. But computing the three-dimensional (3D) architectures of the proteins would currently require a huge amount of time and finances, so it is beneficial to craft software codes to foretell the 3D architecture of a protein. Though the sequence data of almost all proteins is accessible, it is still not possible to deduce precise de novo presaging of their 3D architectures. Of late, due to the impressive power of attribute extrication, DL approaches continue to be implemented to foretell the secondary structure [28], backbone torsion angle [29] and residue contacts of proteins [30]. To cite an example, the DL approach capable of amalgamating one-dimensional (1D) with two-dimensional (2D) “Combinatorial Neural Network (CNN)” to foretell the residue contacts defeated other approaches in “12th Community Wide Experiment on the Critical Assessment of Techniques for Protein Structure Prediction (CASP12)” [30,31]. The architecture of DL may flawlessly learn the linkages among the sequence and the structure via attribute extraction. At present, accurately foretelling 3D architectures of proteins is still an unrealized goal. Hence, the DL approach has demonstrated tremendous potential in advancing the development in this arena.

#### 2.1.2. Prognostication of Protein–Protein Interactions

The protein–protein interactions (PPIs) are vital for various biological systems and may be associated with many disorders [32,33]. A PPIs database, viz., the “Search Tool for the Retrieval of Interacting Genes/Proteins (STRING)” database, hoards close to 1.4 billion PPIs imported from both experimental as well as bioinformatics schemes via literature curation [34]. Moreover, STRING also hoards computationally presaged interactions culled from:

- text mining of scientific documents,
- interactions estimated from genomic attributes, and
- interactions conveyed from model organisms, depending upon orthology.

All approximated/imported interactions are gauged against a common reference of functional association as commentated by the “Kyoto Encyclopedia of Genes and Genomes (KEGG)”.

The PPI interface is represented as the protein–protein association loci made up of multiple residues [35]. It has the potential to usher in a new group of drug targets that are in contrast to the conventional drug targets like ion channels, G-protein coupled receptors (GPCRs), kinases, and nuclear receptors [36,37]. To explain, there exist 1756 non-peptide inhibitors across 18 families of PPIs recorded in the “inhibitors of protein–protein Database (iPPI-DB)” [38]. Being a fresh group of targets, PPIs will expand the target space and boost the advancement of the small molecule compounds [39]. In contrast to conventional approaches, targeting PPIs may lessen the adverse effects as it enhances the biological specificity of regulatory actions [40]. For example, compound DC_AC50 can block copper ion movement inside cells by associating with the copper-transfer mechanisms, and curb tumor cell multiplication selectively without concomitantly interfering with the usual somatic cell durability [41].

To accomplish the concept of drug design dependent upon the architecture of the protein–protein complex, it is imperative to scrutinize the PPI interface. Regrettably, in a great many instances, the precise PPI information is scant [42], thereby spawning a multitude of computational schemes for foretelling the PPI interface. The approach relying on the template is straightforward and highly dependable due to the attribute preservation of PPI interfaces [43]. To cite an exemplar, “eFindSite” [44], a web server for PPI interfaces forecasting, engages template-dependent, residue-dependent, and sequence-dependent determinants to promote “Support Vector Machine (SVM)”, and “Naïve Bayes Classifiers (NBC)” paradigms. According to the doctrine of complementarity, the protein–protein docking processes (e.g., “ZDOCK” [45] and “SymmDock” [46]) can be utilized to foretell the PPI interface when the architecture of two interactive proteins is accessible [47]. Of these approaches, the vexing problem is how to foretell the conformational shifts when two unassociated proteins combine with each other. DL approaches can extricate the most pertinent sequence attributes to presage PPI interfaces, which exemplifies a noticeable enhancement in comparison to other ML technologies like SVM [48].

Taking note of the sizable buried surface area region (1500–3000 Å^2^) of the interface [33], it is imperative to hunt for the druggable locales or regions on the interface. Detectable hot spots could possibly represent the druggable loci since it furnishes a sizable quantum of binding free energy [35]. “Fragment Docking and Direct Coupling Analysis (FD-DCA)” has been engaged to identify the druggable PPI loci [49]. Researchers initially devised a fragment docking package named “iFitDock”, that could be utilized to scout for the druggable hot spots residing in PPI interfaces. Consequently, the small hot spots were bundled to establish candidate binding loci. Ultimately, the scoring function dependent upon the evolutionary conservative level was engaged to identify the optimized protein–protein binding locales. Collectively, the hot spots residing in the PPI interface have turned out to be encouraging drug targets and it is worthwhile to evolve computational avenues for determining the hot spots and crafting small modulators aiming at PPI interfaces.

#### 2.1.3. Prognosticating Drug–Protein Interactions

Drug–protein interactions (DPIs) play a crucial part in the success of a therapeutic entity. The prognostication of a drug–receptor or a drug–protein association is imperative to comprehend its effectiveness and success, enabling drug repurposing, and thwarts poly-pharmacology [50]. A multitude of AI approaches have been beneficial in the precise prognostication of ligand–protein interactions assuring augmented therapeutic effectiveness [50,51,52,53,54]. A description of a model utilizing the SVM method, incorporating training on about 15,000 protein–ligand interactions, that have been advanced grounded upon primary protein sequences as well as structural features of small compounds to identify nine fresh molecules and their interaction with four vital targets have been documented [55].

A research group took advantage of two RF paradigms to presage achievable DPIs due to the amalgamation of pharmacological as well as chemical information and corroborating them against familiar algorithms, like SVM, with great sensitivity and specificity. Moreover, such approaches were adept at presaging drug–target interactions which may be further widened to capture target–disease as well as target–target interactions, hence hastening the drug discovery mechanism [56]. Espousing the “Synthetic Minority Over-Sampling Technique (SMOST)” and the “Neighborhood Cleaning Rule (NCR)” to gather refined data for the ensuing advancement of iDrugTarget has also been documented. This is a merger of four subpredictors (iDrug-Chl, iDrug-Enz, iDrug-GPCR, and iDrug-NR) for determining associations among a drug and ion channels, enzymes, G-protein-coupled receptors (GPCRs), and nuclear receptors (NR), respectively. Upon correlation with extant predictors via target-jackknife tests, the earlier technique outperformed the latter with respect to both forecast precision and consistency [57].

The power of AI to foretell drug–target associations has also been employed to boost the repurposing of available drugs and obviating poly-pharmacology. Repurposing an already available drug entitles it undeviatingly for Phase II clinical trials [58]. Such strategies curtail financial outlay, as reintroducing an already-available drug costs USD ~8.4 million in comparison with the introduction of a fresh drug entity (USD ~41.3 million) [59]. The ‘Guilt by association’ method may be engaged to foretell the ingenuous interaction between a drug and disease, that is either a knowledge-based or computationally guided interactive grid [60]. For computationally impelled networks, the ML method is extensively employed, which engages methods like SVM, NN, and logistic regression, as well as DL. Algorithms based on logistic regression, like “PREDICT”, “SPACE”, as well as other ML techniques, take into consideration disease-to-disease and drug-to-drug resemblance, the comparability among target compounds, chemical architecture, and gene expression summary at the time of drug repurposing [61].

Cellular network-guided DL technology (“deepDTnet”) has been scrutinized to foretell the therapeutic utility of topotecan, presently utilized as a topoisomerase blocker. This could additionally be employed as the treatment for multiple sclerosis by causing blockade of human retinoic acid receptor-associated orphan receptor-gamma t (ROR-γt) [62]. This package is presently covered by a temporary US patent. Self-Organizing Maps (SOMs) constitute the unsupervised subdivision of ML and are utilized in drug repurposing. SOMs engage a ligand-dependent pathway to determine novel off-targets for a group of drug compounds by training the algorithm on a designated count of molecules with perceived biological actions, that is subsequently utilized for the investigation of various agents [63]. In contemporary practice, Deep Neural Networks (DNN) have been engaged to repurpose extant drugs with established actions towards influenza virus, SARS-CoV, HIV, and drugs which happen to be 3C-like protease blockers. For this, Extended Connectivity FingerPrint (ECFP), Functional-Class Finger-Prints (FCFPs), and a Ghose-Crippen octanol-water partition coefficient (ALogP_- count)” were contemplated to train the AI algorithm. As per the results, it was determined that 13 of the molecules subjected to screening could be advanced for further advancement depending on their cellular toxicity and viral blockade activities [64].

Drug–protein associations may also foretell the probability of poly-pharmacology, referring to the proclivity of a drug compound to bind with various receptors resulting in off-target adverse actions [65]. AI can craft a new compound based on the philosophy of poly-pharmacology and facilitate the origination of safer drug compounds [66]. AI algorithms like SOM, in concert with the enormous databases accessible, may be engaged to connect multiple molecules to many targets and off-targets. Bayesian classifiers and Schoof–Elkies–Atkin (SEA) algorithms could be utilized to provide connections betwixt the pharmacological attributes of drugs and their feasible targets [63].

A research group determined the utility of “KinomeX”, an AI-enabled online algorithmic tool engaging DNNs for the identification of poly-pharmacology of kinases depending on their chemical architectures. This package utilizes DNN trained with ~14,000 bioactivity data points advanced and optimized from > 300 kinases. Hence, this has pragmatic pertinence in analyzing the overall specificity of an agent for the kinase family and certain subfamilies of kinases, hence facilitating in scheming novel chemical alterants. This research group employed NVP-BHG712 as a prototype molecule to foretell its primary targets and in addition its off-targets with justifiable precision [67]. One obvious exemplar is Cyclica’s proteome-screening AI scheme that is cloud-based, christened “Ligand Express”. It can be employed to identify receptors that can associate with a specific small compound (the molecular attributes of which is contained in SMILE string) and yield on- and off-target associations. This aids in comprehending the probable adverse actions of the medicinal molecule [68].

#### 2.1.4. *De Novo* Drug Design

During the last several years, the *de novo* drug design method has been extensively employed to craft drug compounds. The conventional approach to *de novo* drug design has been substituted for by emerging DL paradigms, the earlier one having drawbacks of convoluted synthesis pathways and bothersome augury of the biological effects of the innovated compounds [69]. Computer-guided compound crafting may also offer millions of chemical architectures which could be originated and in addition presage multiple variable pathways for them [70].

Origination and advancement of the “Chematica program” [71], now renamed “Synthia,” has the capacity to cipher a group of axioms into the machine and recommend feasible synthesizing pathways in respect of eight medicinally indispensible targets. Synthia has confirmed efficiency from the point of view of bettering the harvest and curtailing the expenditure. The program is well-suited to catering to substitute synthesizing blueprints for patented items and is conceived to be beneficial in the generation of molecules that are yet to be originated. From an analogous context, DNN emphasizes upon edicts of organic chemistry as well as retrosynthesis, which, along with the help of “Monte-Carlo Tree Searches (MTCS)” and symbolic AI, assist in reaction forecasting and the mechanism of design and unravelling of drugs, that is much more nimble compared to conventional approaches [72,73].

A research group refined a framework where an inflexible forward reaction blueprint was practiced on a set of reactants to generate chemically achievable products having a convincing reaction rate. ML was utilized to analyze the principal product depending upon a score provided by the NNs [74]. A DNN framework termed the “Reinforced Adversarial Neural Computer (RANC)” based on Reinforcement Learning (RL) was utilized for small organic molecule *de novo* design. This system underwent training with compounds characterized as SMILES strings. This then originated compounds with preordained chemical descriptors with respect to MW, logP, and Topological Polar Surface Area (TPSA). RANC was probed against one different platform, ORGANIC, where RANC performed better in originating unique structures free from notable attenuation of the length of their structure [75].

RNN was also dependent upon the “Long Short-Term Memory (LSTM)” associated with compounds garnered from the ChEMBL database and introduced as SMILES strings. Such a module was engaged to originate a varied library of compounds for Virtual Screening (VS). Such a method was targeted to solicit innovative compounds for a specific target, like 5-HT2A receptor, *Staphylococcus aureus*, as well as *Plasmodium falciparum* target sites [76].

The Reinforcement Learning for Structural Evolution (RLSE) program dealing with *de novo* drug synthesis by engaging generative as well as predictive DNNs to evolve fresh molecules has been documented. For this, the generative paradigm delivers more exclusive molecules with respect to SMILE strings grounded upon a stack memory, although the predictive approaches are utilized to presage the attributes of the originated molecule [77]. Efforts are underway to harness the generative AI paradigm to craft retinoid X as well as PPAR agonist compounds, with coveted therapeutic outcomes free from necessitating baffling regulations. Five molecules have been successfully originated, four of these have exhibited convincing modulatory actions in cell assays, hence underscoring the utility of generative AI in fresh compound origination [78]. The performance of AI in the *de novo* modeling of compounds can be salutary to the pharmaceutical industry due to its multifarious benefits, such as making provision for online learning and concurrent refinement of the previously-learned data and proposing feasible synthesis pathways for compounds, with consequent brisk lead conception and progression [76,79].

### 2.2. Hit Discovery

#### 2.2.1. Drug Repurposing

Drug repurposing, also known as drug repositioning, is interpreted as the method to identify ingenious therapeutic applications of the approved drugs [80,81], which can shorten the period and perils of drug advancement [80]. Drug repurposing is achievable since many drugs may have numerous targets [82] and the targets may elicit their varied actions, which exemplifies the high heterogeneity of drug-disease association. To cite an example, metformin, which was ratified for the management of type two diabetes, may prolong lifespan [83,84,85].

Drugs and diseases are two fundamental components related to repurposing a drug. Auxiliary aspects are also associated with drug repurposing, like targets for drugs and genes for diseases. Owing to the multifariousness of the associations, network scrutiny may be engaged to portray the relationships among these aspects [81]. For the purpose of drug design, there happen to be nine types of meaningful networks: gene regulatory, metabolic, protein–protein, drug–target, drug–drug, drug–disease, target–disease, drug–adverse effect, and disease–disease networks [81]. The primary assumption of the network-dependent scheme is that the analogous drugs frequently possess comparable targets or activities [86]. The data contained in the singleton network is restricted and fractional, hence it is imperative to merge multiple networks to generate the conglomerate network for repositioning one drug. Specifically, it is critical to integrate drug repurposing with the drug target forecasting, as the target could be conceived as a connection from the medication to the affliction. DTINet, a diversified network harmonizing the information of numerous networks via the network diffusion algorithm and the dimensionality reduction methodology, was utilized to foretell the fresh target and therapeutic niche [87]. To cite an exemplar, this approach proposed that alendronate, chlorpropamide, and telmisartan could possess novel cyclooxygenase blocking actions. These activities were subsequently substantiated experimentally by assessing the generation of proinflammatory components, and these three molecules thus furnish high fidelity hits for forestalling inflammation.

#### 2.2.2. Virtual Screening (VS)

Virtual screening implies the implementation of algorithm and software to identify bioactive molecules (hits) from in-house compound assemblage or commercial chemical libraries, that offer a hugely efficient scheme to unravel novel hits and refine out molecules with disadvantageous scaffolds during the early stages of drug development [6]. Virtual screening approaches consist of docking-based, pharmacophore-based [88], similarity searching [89], and ML schemes [90]. Broadly speaking, these approaches can be allocated into two types of virtual screening: structure-based and ligand-based. Molecular docking has been extensively applied when the target protein 3D architecture is accessible [91]. Though numerous successful implementations of docking-aided virtual screening have been established [92], there remain glaring disadvantages of this technique. For instance, scoring function of docking is unable to foretell binding affinities precisely due to incomplete attention to solvation and entropic aspects [93], and the protein flexibility renders the issue even more convoluted [91]. Furthermore, as most docking approaches only account for binding affinities and overlook other parameters like the residence period [94], the docking score is not an optimal clue for drug effectiveness and the false positive frequency of the docking-associated VS is large [91,95].

Contrary to the docking-related virtual screening schemes, the ligand-based virtual screening approaches do not bank on the 3D protein architectural data. They attempt to correlate the molecular attributes (descriptors) with bioactivity classes [6]. Contextually, ML algorithms like SVMs have been often utilized for virtual screening [7,90,96], which has demonstrated significant yields (ratio of presaged known hits) and decremented false-hit frequencies concomitantly (false hit in anticipated hits) [97]. Of late, DL approaches have been tested in VS attributable to their incredible classification capabilities, robust feature extrication power and small generalization error [10,98]. For instance, scanty presence of the active moieties in the general database customarily exhausts a substantial quantum of search duration at VS [77,97]. To provide a solution to this roadblock, a LSTM network scheme grounded on the analogy among natural language and the Simplified Molecular Input Line Entry Specification (SMILES) was implemented to engender targeted molecule libraries with compounds comparable with the training compounds [77]. The new molecular libraries engendered by Recurrent Neural Networks (RNNs) can be probed with ML algorithms like Deep Neural Networks (DNNs) and Gradient Boosting Trees (GBTs). Likewise, due to the compelling generative capacity, an Adversarial AutoEncoder (AAE) model was trained dependent upon the NCI-60 cell line assay information [99], which can then be implemented to originate molecular fingerprints for exploring promising anticancer agents [100].

#### 2.2.3. Activity Scoring

As stated earlier, the fundamental attribute of molecular docking happens to be the scoring function, which is crafted to appraise the binding proclivities of the drug-like moieties for a target of relevance [101]. Due to the robust nonlinear mapping capacity, ML- associated scores display improved method execution by extricating numerous attributes efficiently, like the geometric attributes, chemical characteristics as well as physical force field traits [102]. Such scores may be contemplated as data-impelled black box paradigms as they foretell the binding affinity or ligand-protein binding association from experimental information directly and bypass the consideration of the convoluted physical function associated with docking [103]. ML algorithms such as Random Forest (RF) and Support Vector Machine (SVM) can be engaged to enhance the performance of scoring function. As an instance, in place of utilizing the linear additive acceptance of energy terms, an SVM paradigm chronicled the nonlinear association among the particular energy terms borrowed from the docking algorithm “eHiTS” and experimental binding affinity information exhibited enhanced screening power in addition to scoring capability [104,105]. Wang and Zhang documented a ΔvinaRF parameterization correction approach amalgamating RF with AutoDock scoring function [106], which displayed an admirable performance correlated with GlideScore XP [107]. In recent times, owing to the exemplary performance of Convolutional Neural Networks (CNN) in the vista of image refinement [108], several researchers have endeavored to utilize CNN to glean the attributes from protein-ligand interactions image in order to foretell the protein-ligand affinity. A research group utilized a 3D graph CNN algorithm to foretell ligand-protein binding proclivities [109], which provided data that the anticipated binding tendencies had acceptable correlation with experimental information within the datasets. The true capability of DL rests in its competence to grasp convoluted and abstruse attributes from elemental and primeval visage. Hence, it is vital to portray the basic aspects of the compound protein complex like the atom types, atom charge, atom distance and amino types [108]. Deep VS, an algorithmic scheme grounded on CNN, can grasp the abstruse attributes from the key characteristics (the atom frame of reference) and it performed better than the conventional docking algorithms like Internal Coordinate Mechanics (ICM) [109], and GLIDE SP [110] on the Directory of Useful Decoys (DUD) in the context of “area under the curve of receiver operating characteristic (AUCROC)” and enrichment factor [111]. In principle, the CNN approach foretells the binding tendencies by gleaning the characteristics in the protein-ligand association image, which is quite analogous to a knowledge-dependent scoring function coupled with augmented prognosticative prowess.

### 2.3. Hit-to-Lead Optimization

#### 2.3.1. Quantitative Structure-Activity Relationship (QSAR)/Quantitative Structure-Property Relationship (QSPR) and Structure-Aided Modelling with AI

In the course of hit-to-lead refinement, QSAR analysis could be utilized to determine the potent leading molecules from a collection of hits analogues by foretelling biological effects of related molecules. QSAR especially points to the utility of mathematical techniques for determining the quantitative mapping of the architectural or physicochemical attributes of molecules and their affiliated biological effects [112]. QSAR investigation primarily encompasses data capture, selection, and origination of molecular descriptors, formulation of mathematical paradigms, and evaluation and analysis of models, as well as engagement of models [113]. Of these, the crucial points are the depiction of the chemical architecture and the mathematical paradigm capturing QSAR. Following choice of the descriptors, it is imperative to identify a relevant mathematical algorithm to match the structure-activity co-relationship. In 1964, Hansch et al. advanced the famous “Hansch Equation” that ingeniously employed linear regression techniques in relation to physicochemical descriptors (the hydrophobic parameter, the electronic parameter and the steric parameter) for narrating the 2D structure-activity correlation, leading to the era for QSAR evaluation [114]. In the same year, a research group developed the “Free-Wilson” technique to narrate the association between the chemical architecture and bioactivity depending upon the assumption that the addition of substituents to the actions of the compound is supplementary [115]. In contrast to the Hansch technique, there is no need in the Free-Wilson approach for the physiochemical criteria and it can directly foretell the biological actions from the chemical architecture by ciphering the chemical framework. Owing to the progress of ML algorithms, numerous approaches have subsequently been utilized to engineer mathematical paradigms [116,117,118], like RF and SVM. Of late, DL algorithms have undergone integration with QSAR modeling due to the capacity of handling disparate chemical attributes coupled with the worthiness of extricating attributes in an automated fashion. George Dahl’s team triumphed in the Merck Molecular Activity Challenge (a Kaggle championship event conducted in 2012 and concerned with QSAR problems), by the composite (ensemble) paradigms comprising the multi-task DNN, Gaussian progress regression as well as gradient boosting machine techniques [8]. Energized by the Kaggle championship outcomes, Dahl et al. went on to methodically investigate the multi-task DNN and his output had demonstrated that multi-task DNN outclassed single-task neural network approach as the multitask approach may recognize generic characteristics by allocating specifications of varied but allied assignments [119]. A research group amalgamated multi-task neural networks within the “DeepChem” platform that assisted the engagement of multi-task neural network algorithms in relation to drug advancement [120]. They also assessed the execution efficiency and identified the fact that multi-task deep networks were quite powerful and superior to random forests (RFs) on diverse assignments. Another research group engaged the DNN with Canvas descriptors to construct the classification and regression paradigm to foretell the binding proclivities of the human β-secretase 1 (hBACE-1) blockers [121]. On the validation set, this DNN technique engendered robust classification capacity with a certainty of 0.82 and, in addition, displayed favorable regression capability with the coefficient of determination (*R*2), and mean absolute error (MAE) of 0.74 and 0.52, respectively. Also, their outcomes have demonstrated that the DNN approach coupled with 2D descriptors offered superior results compared to the force-field-dependent schema (e.g., CoMFA), which is partly because of the compelling generalization proficiency of DLapproaches. Evidently, DL-aided QSAR algorithms with augmented activity prediction accomplishment will exert a more salutary role in the subsequent hit-to-lead refinement techniques.

#### 2.3.2. Generative Schemes for De Novo Drug Design with AI

De novo drug design relates to engendering fresh chemical moieties to inflect the target of relevance [122]. The conventional de novo design approaches like the fragment-aided method can engender fresh moieties from scratch. But most of these are onerous to originate owing to the complexity and inapplicability of the compound architecture [123]. Moreover, it is difficult to assess their biological effects because of the demerits of scoring functions ascribed to in an earlier section.

Due to the compelling generative capacity and learning capability, DL algorithms have been engaged to automatically engender new architectures with several coveted attributes [124]. One research group advanced the deep reinforcement learning approach to refine the RNN to originate the compounds with anticipated biological effects [125]. The “Simplified Molecular-Input Line-Entry System (SMILES)” structures of molecules culled from ChEMBL were employed to train the “Recurrent Neural Network (RNN)” for obtaining the syntax of SMILES, and the RNN could originate the molecules by representation from the conditional probability distribution related to the training group. With reference to reinforcement learning, Agents are the decision-takers who execute actions in the presence of specified conditions. When an Agent’s action results in a positive reward, the Agent’s proclivity of producing this action will be augmented [126]. SVM has been engaged to promote the action protocol for receiving the high anticipated reward from activity scoring depending upon the ligands in the training dataset. In the instance of selection of RNN along with the deep reinforcement learning (DRL) paradigm to originate agents for dopamine receptor type 2, > 95% of the architectures were presaged to be biologically active from the SVM scoring function.

An added utility of generation paradigms with DL is the engagement of auto-encoders to spawn novel molecules. A research group amalgamated the Variational AutoEncoder (VAE) with the Multilayer Perceptron (MLP) to originate fresh molecules with salutary attributes in an automated manner [127]. The network comprised of three components: the encoder, the decoder, and the predictor. While the encoder converts disconnected SMILES strings into uninterrupted vectors in latent space, the decoder can convert these vectors in reverse order to the disconnected SMILES strings. Engagement of MLPs is done to foretell the attributes of the compounds, and the gradient-enabled refinement can be utilized to determine the uninterrupted vectors with high predictive potential of the characteristic. Due to the durability of the vector depiction in the latent space, the gradient-enabled optimization united with Bayesian reasoning can be employed to promptly determine the compounds with coveted attributes. The paradigm has the power to originate a human-intelligible chemical architecture with greater predictive effects in an automatic fashion. But it also resulted in several instances of fallacious SMILES production which are unrelated to legitimate chemical architectures. To surmount this problem, a group engaged the grammar VAE to render the result more efficient by specified SMILES syntax [128]. Very recently, a group popularized an AAE scheme named druGAN to originate molecular fingerprints, that outclassed the VAE paradigm in the context of the reconstruction error, generation power, and attribute extrication potential [129].

To assess if an engendered molecule is synthetically attainable, the group of Coley et al. specified a synthetic complexity metrics by tuning-up a neural network algorithmic scheme based on a reaction database [130]. The axiom for calculating synthetic intricacy is that the synthetic reaction is a system that will raise complexity of the reacting agent. In case of a synthetic reaction, it implies that the product complexity score must exceed that of the reacting agents. Hence, the aforementioned group ciphered a chemical reaction into many (reactant, product) combination duets and undertook to formulate a scoring function for depicting the inequality association among reactant complexity and product complexity. As the neural networks possess compelling function approximation capabilities [131], this group utilized 22 million (reactant, product) duets in order to train the neural network in order to account for scoring function learning, with their outputs elucidating that the learned function (SCScore) might well chronicle the intricacy enhancement in the synthesis method. This scheme will facilitate chemists in achieving the inverse synthetic analysis and in addition assist them set aside improbable molecules in drug design by appraising the synthetic intricacies.

#### 2.3.3. Automated Chemical Synthesis Planning with AI

##### Foretelling the Retrosynthesis Roadmap

Retrosynthesis is a refined scheme for conniving organic synthesis. In concert with the progress of AI, this assignment can now be undertaken much more skillfully [73,74,132,133,134]. Following virtual screening of a molecule for its conceivable biological effects and toxicity attributes, the hunt for an efficient chemical synthesis roadmap to generate the drug candidate is initiated. This task is often imposing and unrefined. In spite of awareness of hundreds of thousands of conversion steps, there is no assurance that novel molecules may be expertly generated owing to novel structural characteristics or opposing reactivities [135].

Retrosynthesis analysis recursively probes for ‘backward’ reaction pathways until a group of less complex, accessible precursor compounds are accomplished [74]. As retrosynthesis pathway presage engages successive scissions of the lead molecule at numerous locations, Monte Carlo Tree Search (MCTS) [136] is the algorithm of choice for executing branch choices. Monte Carlo simulations implement random search steps with no branching till the time an optimal result is reached. Hitherto, software programs for Computer-Assisted Synthesis Planning (CASP) [137,138] were advanced to facilitate retrosynthesis scrutiny, but missed gaining full approval with the chemists. Such algorithms insist that human insight is integrated within executable schemes, but formalization of chemistry with the aid of manual ciphering will not add up to exponentially multiplying knowledge, and the outputs fetched from reaction databases were most frequently devoid of chemical intelligence [74]. ML algorithms trained on provisional data may now be engaged; (i) to foretell the chances of a conversion at a distinct branching location, and (ii) to pilot the choice of the random steps. For individual conversion steps, the compound (or an intermediary molecule) could be related to distinct forerunner molecules through preordained conversion axioms. Training of AI packages can be done from the scientific documentation with regard to the yields and expenses of these conversion statutes, and the AI can then presage the best suitable retrosynthesis conversion roadmap for a selected compound.

A recently documented 3N-Monte Carlo Tree Search (3N-MCTS) technique [74] incorporates three disparate neural networks incorporating MCTS to engender a roadmap for Critical Assessment of Protein Structure Prediction (CASP). CASP exemplifies the latest state-of-the-art in modelling protein structure following up from amino acid sequence. Each network executes a separate assignment: (i) an expansion node; (ii) a rollout node; and (iii) an update node. For the expansion node, the algorithmic process probes for fresh prospects for modifying the compound (or an intermediary molecule), retrospectively. This embodies an ‘in-scope’ protocol where the workability of a modification is appraised depending upon 12.4 million conversion axioms from the scientific documentation [139]. In order to foretell the best modification for the compound (or intermediary molecule) available, and hence pilot the selection of expansion routes, training the neural networks is imperative. As the literature copiously contains positive data, a modification is deemed less workable if its reverse reaction provides a high yield. Also, choosing high-yielding conversions also facilitates in excluding the chances of by-products [74]. For the rollout node, the ‘in-scope’ protocol is analogous to that in the expansion node, with the exception that only commonly documented conversion axioms are utilized. This approach permits a gradual and meticulous hunt for the optimized conversion alternatives at the time of the expansion period, but swifter scrutiny of position values during the rollout phase [140]. For the update node, the appraisal of a distinct roadmap is amalgamated into the search tree. In the case of a molecule deposited for retrosynthesis scrutiny, these nodes are executed repetitively to explore for conversions with the greatest scores. The latter can ultimately determine feasible precursors associated with the complete reaction pathway [74].

Along with the determination of a reaction route, the time expended to arrive at a solution is also a vital parameter of algorithm execution. A time constraint could be imposed to evaluate the fraction of issues which an algorithm is able to tackle. The achievement of MCTS on the test group of compounds was better than various available software. MCTS tackled 80% of retrosynthesis issues when a 5s per molecule time constraint was imposed [74], and the frequency of arriving at a solution can surpass 90% if the time constraint is extended to 60 s. More attractively, the speed for each molecule for 3N-MCTS is 20-times swifter compared to the conventional Monte Carlo approach [74].

##### Prediction of Yield of Reaction and Understanding of Reaction Scheme

AI packages can not only depict synthesis pathways but in addition can adequately foretell the products with yields of organic reactions depending upon the molecular characteristics of the reactants. Previously, foretelling the result of complex chemical reactions was associated with a sizable bottleneck [133]. Quantum chemistry methods, for instance, the “Hartree–Fock Method” [134], semi-empirical processes (AM1, PM3), and density functional theory, will possibly surmount such an obstacle, and in various scenarios the result of tests can be optimally simulated in silico. Many studies engaging AI schemes to automate, advance, and establish yield prediction have of late been documented for this sector [141,142,143,144], and Doyle and Dreher testified that ML can be engaged to foretell the returns of a Buchwald–Hartwig coupling reaction [145]. The aforementioned reaction engenders carbon–nitrogen bonds amid aryl halides and amines, utilizing the catalyst palladium, and has been extensively practiced for the total synthesis of pharmaceuticals where aryl amine bonds are pervasive. For this scenario, the vibrational frequencies and dipole moments computed by quantum chemistry were considered as descriptors, and the ultimate product returns from a provided group of reactants were generated by high-throughput experimental syntheses. A RF scheme was then utilized to probe the association among the input descriptors and product returns [133]. At the time of utilizing reactant variants, the algorithm also presaged the yields of other anticipated products with great precision [145].

##### Synthesis Methods Digitized and Standardized

There are enterprising plans to harness AI to mechanize chemical syntheses with limited manual processes. Recently proven technologies, like the ‘solid phase’ scheme where the growing polymer chain is attached to an insoluble matrix, have mechanized the generation of many classes of agents inclusive of peptides [146] as well as oligonucleotides [147]. But these depend on distinct protocols due to the shortage of standardized digital mechanization methods for computer monitoring of chemical reactions, and no universal software is present for computational governance of chemical operation systems [148] (Table 1). The “Chemputer platform” [149] was newly advanced as a standard benchmark which integrated codified standard recipes, or chemical codes, for compound synthesis. The scheme was executed with the “Chempiler program” [149], one that obtains codified methods from a scripting language “Chemical Assembly (ChASM)”, which also regulates distinct low-level execution rules for the modules that make up the structure of the robotic system. ChASM draws upon a chemical descriptive language (ΧDL) which exclusively and methodically amasses the complete obligatory information for a synthesis operation [149]. The physical modules (e.g., the source flask and the target flask) and their network arrangement and portrayal are depicted as a directed graph by engaging an open-source markup language termed “GraphML” [150]. With “GraphML”, “Chempiler” is capable of governing the robotic procedures in a manner that users can exactly execute chemical syntheses without manual restructuring. This system had been approved by the fruitful synthesis of three pharmaceutical molecules: diphenhydramine hydrochloride, rufinamide, and sildenafil, bereft of any human interference, and with outputs and pureness of products commensurate with or superior to those accomplished manually [149]. This work epitomizes a leap towards the full mechanization of bench-scale chemistry with supplementary benefits of augmented replicability, security, and availability of complex compounds.

##### AI-Enabled Mechanized Reaction Space Sampling

Synthesis robots in conjunction with AI can also be utilized to examine the uncharted reaction space. Of late, Leroy Cronin and group employed a synthesis robot to execute reactions with non-premeditated substrates where the choice of substrates was communicated as a vector depiction that was accepted as the input for the SVM model [168]. Employing mechanized reaction appraisal of the sample with infrared (IR) and NMR spectroscopy, the model implemented a dual categorization of the reactivity of each substrate duo. The reaction database was then revised appropriately, and a Linear Discriminant Analysis (LDA) [169] algorithm was trained on the chemical space to foretell the possibility of the reactions left. LDA explores a linear amalgamation of chemical characteristics that foretell whether a reaction occurs or does not occur. This repetitive workflow was determined to foretell the reactivity of roughly 1000 reaction combinations demonstrating ~ 80% accuracy employing real-time information from only a few experiments [170]. When this ‘self-driving’ methodology was further brought to bear upon Suzuki–Miyaura reactions [171], the predicted reactive combinations were tracked manually by a chemist, with subsequent uncovering of four hitherto uncharted reactions. Following comparison with the reactants and products of millions of reactions, the Tanimoto similarity scores [172] of the four already unknown reactions were determined to be in the top 10 percentile, proposing the concept that these reactions are separate from others selected at random [170]. This method is a crucial step in the digitization of chemistry that might permit real-time exploration of chemical space to be an actuality, and facilitate chemists in uncovering fresh drug leads in a more time- and cost-effective fashion.

### 2.4. In Silico Assessment of ADME/T Attributes

#### 2.4.1. Physico-Chemical Characteristics

Early recognition of compounds with undesirable physico-chemical characteristics in a drug discovery channel indubitably decreases the possibility of loss. Various DL-based algorithms have been advanced on this issue [173]. Duvenaud et al. employed the CNN–ANN to foretell the solubility by extricating data straight from the molecular graph with a compelling predictive capability outcome (MAE is 0.53 ± 0.07) [158]. The high point of this approach rests in its tractability. As an instance, the pieces endowing molecule solubility like hydrophilic R-OH substituent can be realized by model backtracking. Encouraged by Duvenaud’s effort, the research group of Coley et al. used a tensor-dependent convolutional inlay of associated molecular graphs schema to foretell the molecular aqueous solubility, which outclassed Duvenaud’s scheme (MAE is 0.424 ± 0.005) [130]. The scheme used a molecular tensor assimilating the bond-level as well as atom-level characteristics to chronicle associated molecular graph. In comparison with Duvenaud’s paradigm, Coley’s scheme employed greater atom-level data to foretell the aqueous solubility of the compound.

Due to the fact that a strong relationship was identified among oral drug absorption and Caco-2 permeability coefficient (P_app_) [174,175], presaging the candidate drug P_app_ performs a crucial role in appraising the pharmacokinetic characteristics of candidate agents. 1272 molecules have been culled with Caco-2 permeability information by utilizing Boosting, Support Vector Machine (SVM) regression, Partial Least Squares (PLS), and Multiple Linear Regression (MLR) to establish the presaging algorithms with 30 descriptors [176]. The Boosting model displayed the best outcomes with compelling predictive capability (R2 = 0.81, root mean square error (RMSE) = 0.31) for the test compound group, and this model rigorously adopted the Organization for Economic Co-operation and Development (OECD) axioms pertinent to QSAR/QSPR [177]. A train of processes adhering to the OECD tenets assure the coherence and reliability of the paradigm.

#### 2.4.2. Pharmacokinetic Parameters (Absorption, Distribution, Biotransformation and Excretion)

Drug absorption is the mechanism by which medications get into the bloodstream from the administration location. Bioavailability is a critical pharmacokinetic attribute that embodies the quantum of absorption. Foretelling the bioavailability of a compound can facilitate the medicinal chemist to refine its absorption characteristics. A research group garnered a dataset containing 1014 compounds and utilized the MLR paradigm to presage bioavailability aided by structural fingerprints as well as molecular attributes [178]. Genetic function approximation method was utilized to determine the choice of molecular attributes employed for process training in an automated manner, and the outcomes provided a compelling predictive accomplishment, the correlation coefficient and RMSE being 0.71 and 0.2355, respectively.

Drug distribution is the method by which drug molecules move in blood to interstitial fluid, as well as intracellular fluid consequent to drug penetration [179]. The steady-state distribution (VDss) of a medication is the ratio between its dose in vivo to its steady-state plasma concentration (CPss). The VDss signifies the measure to which a medicinal molecule is disseminated in the tissue and happens to be a crucial parameter to appraise drug distribution. Foretelling VDss can facilitate medicinal chemists to implement structural alterations for superior pharmacokinetic characteristics. A research group amassed a dataset containing 1096 molecules and built-up Partial Least Squares (PLS) and Random Forest (RF) paradigms to foretell VDss [180]. The presage outcomes of their algorithm on the external test group were disappointing, as only about 50% of the molecules were within two-fold error. Ostensibly, it is problematic to presage VDss value purely from molecular architectural data as there happen to be multiple unidentified parameters which may influence VDss.

Following the administration of the drug into the body, it will initially encounter the metabolic process with resultant attrition of drug effects, or, in a few instances, origination of toxic metabolites. Foretelling the location of biotransformation with great precision can facilitate the structural refinement for assuring the metabolic endurance of the moiety. A colossal quantum of information associated with drug metabolism has been culled, and multiple ML schemes have been utilized to foretell the loci where molecules are bio-transformed by disparate metabolic enzymes, like cytochrome P450s (CYP450s), aldehyde oxidase, and Uridine 5′ diphosphoglucuronosyltransferases (UGTs). As an instance, on the basis of a neural network approach, “XenoSite” [181] can deliver the determination of the location of small compounds biotransformed by CYP450s with a gross accuracy of 87% [182]. Moreover, the “XenoSite”scheme also employs a neural network trained upon a vast database of UGT biotransformation to foretell UGT loci of the molecule biotransformation [183].

Drug excretion is the mechanism by which medications and their bio-transformed metabolites are disposed of from the system. Bio-transformed metabolites of medications are generally water-soluble and can be readily discarded from the body while some drugs can be directly disposed of without biotransformation [184]. Lombardo et al., employed the Principal Components Analysis (PCA) approach to foretell primary clearance pathway and the algorithm exhibited good discrimination outcomes among various approaches, with a predictive precision of 84% [185]. Depending upon the elimination process prediction paradigm, this group engaged the PLS algorithm to foretell the gross human clearance and the PLS paradigm worked satisfactorily and was comparable to animal scaling approaches.

#### 2.4.3. Toxicity and the ADME/T Multi-Task Neural Network

In the course of development of fresh drugs, pre-clinical and clinical toxicity accounts for the reduction of roughly 33% of leading moieties [186]. Hence, foretelling the toxic effects of compounds is invaluable in facilitating the refinement of lead moieties and trimming the hazard of loss in the course of drug development. Conventionally, drug toxicity characteristics (e.g., hepatotoxicity and nephrotoxicity) are foretold by axiom-dependent expert knowledge and architectural flags, which appear to engender false positives and are incapable of broadly encapsulating all mandatory structural characteristics. Currently, owing to the capability of handling varied chemical entities and the virtue of extracting attributes in an automated fashion, the DL algorithms churn out compelling performance on toxicity presage. As an instance, based upon the “Molecular Graph Encoding-Convolutional Neural Networks (MGE–CNN)”, Xu et al. crafted an acute oral toxicity prediction paradigm, and the presage outcomes were superior to the hitherto documented approaches based on SVM [187]. In the MGE–CNN scheme, the molecular ciphering, attribute extrication and model building is executed by methods analogous to the neural networks training. Also, the MGE–CNN algorithmic scheme is quite adjustable since molecular fingerprints can be tailored as per the specific issues. A research group correlated the toxicological characteristics of fingerprints back to atomic levels and gathered several highlighted pieces that conform to structural flags characterized in the “ToxAlerts” [188]. Hence, due to analogy with Duvenaud’s model, this paradigm by Xu et al. is also explicable. Another group originated a multi-task DNN algorithm named “DeepTox” to foretell the toxic effects and the “DeepTox” system certainly outclassed numerous contenders in the Tox21 competition [163]. By accepting the identical criteria, the multi-task neural network algorithm was trained to foretell numerous disparate discrete assignments that are strikingly connected. In comparison to single-task neural network, the execution of multi-task neural network is customarily superior owing to sharing of the criteria of various assignments in facilitating the multi-task algorithm for ingraining added familiar attributes.

Pharmacokinetic processes (drug absorption, distribution, biotransformation, excretion) and drug toxicity in the human system have some congruity and the multitasking neural networks can enhance the predictive capability of such assignments. ADME/T experimental datasets of Vertex Pharmaceuticals have been utilized to match the capabilities of the single-task and multi-task neural networks, and their outcomes implied that multi-task algorithms would yield superior results as anticipated [189].

## 3. Machine Learning Schemes and Usable Algorithms for Drug Design Scenarios

The representation of molecules has been of interest to scientists since the nineteenth century. Traditionally, molecules are represented as structure diagrams with bonds and atoms, and this is likely the representation most people cognize when they contemplate about molecules. But, alternative representations are imperative for the computational processing of chemical structures in cheminformatics. The advent of computers led to the development of a wide array of machine-readable chemical representations. Computers permitted the rapid digital storage and querying of compounds and their structures, swift modifications of digital data, and augmented physical storage efficiency. Algorithms were implemented to visualize compounds as 2D depictions and the computational visualization of compounds in 3D space was popularized with the advent of specialized programs.

The lead optimization step of drug discovery is fundamentally a low-data problem. When biological studies provide proof that a particular molecule can modulate essential pathways to achieve therapeutic activity, the discovered molecule often fails as a potential drug for numerous reasons including toxicity, low activity, or low solubility. The central problem of small-molecule-based drug-discovery is to refine the candidate molecule by locating analogue molecules with enhanced pharmaceutical activity and reduced risks to the patient. Yet, with only a small amount of biological data available on the candidate and related molecules, it is challenging to form accurate predictions for novel compounds. Recent work has established that standard ML techniques such as random forests and simple deep-networks are capable of learning meaningful chemical information from only a few hundred compounds. Other recent advances in ML have demonstrated that in some circumstances, nontrivial predictors may be learned from only a few data points. These methods work by using related data to learn a meaningful distance metric over the space of possible inputs. This sophisticated metric is used to compare new data points to the limited available data and subsequently predict properties of these new data points. More broadly, these techniques are termed as “one-shot learning” methods. In ML, generalization usually refers to the ability of an algorithm to be effective across various inputs. It means that the ML model does not encounter performance degradation on the new inputs from the same distribution of the training data. Cross-validation (CV) is a technique for evaluating a ML model and testing its performance. CV is commonly used in applied ML tasks. It helps to compare and select an appropriate model for the specific predictive modeling problem. CV is easy to understand, easy to implement, and it tends to have a lower bias than other methods used to count the model’s efficiency scores. All this makes cross-validation a powerful tool for selecting the best model for the specific task. Despite the fact that DL models outclass various conventional ML algorithms, they still invoke many more parameters and unrelated architectures, which leads to several problems during training, specifically in the situations when the samples are inadequate or the feature matrix is meagre. This section of the review details the aforementioned drug design approaches utilizing ML algorithms. Many open-source execution platforms for AI-facilitated drug design paradigms have been outlined in Table 1.

### 3.1. Approaches for Molecular Depiction

Molecular fingerprints, numbers, ASCII strings, and graphs that depict the compounds may be utilized as the input attributes of ML algorithms for drug design. Such molecular fingerprints cipher the molecular features as a sequence of binary bits (“1” expressing that the molecular feature prevails, and “0” signifies that the molecular feature is nonexistent). In the arena of drug design, molecular fingerprints are continually employed to foretell compound characteristics and compute molecular resemblance since it is a straightforward and powerful approach to express the compounds. Currently, the molecular fingerprints often utilized as the neural network inputs are structure-based 2D molecular fingerprints, like the Molecular ACCess System (MACCS) [190], the Extended-Connectivity Fingerprint (ECFP) [191], the Functional Class Fingerprint (FCFP) and the Molprint2D [192]. As an instance, MACCS has been engaged in training an Adversarial AutoEncoder (AAE) algorithm to hunt for anti-neoplastic moieties [105].

Chemists have long employed 2D molecular graphs to depict molecular architectures and scrutinize molecular characteristics qualitatively. Strikingly, the progress of AI renders it feasible to compute this mechanism. CNN is a compelling engine to extricate characteristics from the molecular graph in an automated manner that can be utilized for engendering compound depiction in presaging of bioactivity [193], toxicity [187], physicochemical attributes [158] and protein-ligand affinity [113]. In comparison to ECFP, the graph-convolutional approaches have been more adaptable as the graph architecture can be tailored depending upon the assigned tests. Moreover, the graph-convolutional architecture is amenable to amalgamation with neural networks so as to foretell the molecular attributes, rendering the training mechanism, molecular attribute elicitation and model development accomplishment concurrently. The molecular graph CNN fingerprints comprise Duvenaud’s graph convolutional fingerprints grounded on atomic radiation technique [158], Kearnes’ graph convolutional fingerprints established upon atoms, bonds and pairwise interconnections [194], and Coley’s graph convolutional fingerprints established upon the molecular tensor. The fundamental tenet of Duvenaud’s graph convolutional fingerprints is akin to the ECFP fingerprints and both these progressively enhance molecular substructures by atomic radiation techniques. Notably, Duvenaud et al., first ciphered atomic attributes (e.g., valence, atomic identity, and number of hydrogens) and bond characteristics into vectors, and then utilized the atomic and bond attribute vectors to build the atomic neighbor attributes to originate the earliest molecular architecture vectors. CNN may be employed to elicit the characteristics from the aforementioned antecedent attribute vectors with individual repetition, and these quantities are then aggregated as the molecular fingerprints. The intrinsic atomic and bond attributes are expert-crafted instead of learning from the molecular graph through the AI process. The superiority of Duvenaud’s graph CNN rests in its strength to engender the molecular fingerprints satisfactorily for a prescribed assignment, and it is explicable as the molecular pieces associated with the distinct molecular characteristics that can be captured by backtracking via the neural network nodes. Such a scheme has been executed in the “DeepChem” toolbox and the outcomes of “MoleculeNet” benchmark assessments indicate that the graph CNN can comprehend fruitful molecular characteristics and it frequently yields superior results compared to other models [195]. Apart from CNN, the recursive neural networks are also amenable for molecular depiction employment. As an instance, Gregor Urban et al. advanced the inner and outer recursive neural networks for graph portrayal of the compound [156]. In comparison with Kearnes’ method, this approach commonly yields superior prediction outcomes on public data groups of the “MoleculeNet” benchmark assignments [195].

The string depictions of small compounds incorporate the Wiswesser Line-formula Notation (WLN) [196], SYBYL line notation (SLN) [197], SMILES [198] and the International Chemical Identifier (InChI) [199]. Out of them, SMILES is more extensively employed backed by multiple software algorithms (like ChemDraw, Cheopy, and RDKit) and databases (e.g., PubChem and ZINC). PubChem (NCBI) happens to be the world’s most comprehensive assortment of freely usable chemical information. It’s the database of chemical molecules and their actions in biological assays. ZINC database (UCSF) is a curated assortment of commercially handy chemical moieties prepared specifically for virtual screening. Recurrent Neural Networks (RNN) can be utilized to comprehend the coding grammar of SMILES [77], which may be transformed to the molecular graph. Moreover, SMILES can be directly employed as an input component of RNN in foretelling the molecular characteristics [200].

Molecular descriptors traditionally relate to the structural or physicochemical attributes of a compound, which can be accessed by molecular ciphering or via typical experiments [201]. The comprehensive characterization of these descriptors has been reviewed elsewhere [202]. The appropriate choice of the descriptors is crucial for ML, which can decrease the computational load, augment the model universalization capability and boost the conduct and characterizability of the algorithm [203]. The usual software to compute molecular descriptors comprises Dragon [204], Cheopy [205], PaDEL [206] and Cinfony [207].

### 3.2. Transfer Learning Engagement for Low Data

The DL schemes have demonstrated a healthy promise in drug design owing to the powerful data mining competence. But the DL approaches generally depend upon a high quantum of training data, that has limited its application customarily. As an instance, with only a restricted quantum of the activity data at one’s disposal, it is problematic to foretell the bioactivity of the fresh compounds since low data is unable to ensnare a sufficient chemical space. A transfer learning approach can be utilized in ironing out issues by taking advantage of extant knowledge acquired from other associated data repositories. It is known that human experts can apply already acquired knowledge to sort out new issues and the capability aids us in solving the vexing issue optimally. A recommendation of AI study is to mimic this capacity by a transfer learning approach [208]. The fundamental tenet of transfer learning is to utilize the knowledge probing from some former exercises to a pertinent target assignment with sparse training data. Moreover, “one-shot learning scheme” has been recommended which alludes to the DL approach that depends upon only a few training items. This is able to pass on information among pertinent, but unrelated assignments by learning a purposeful distance metric [209]. A research group evolved and advanced a one-shot learning scheme that melded the repetitive sophistication of long short-term neural networks engaging the graph CNN for low data training [12,210]. The model performs better than the RF and other techniques on the “Tox21” and “SIDER” dataset. But, when the toxicity data is engaged for training a scheme in foretelling a side effects datafile, it will fully fail as the congruity among the two datasets is quite feeble.

### 3.3. The Process of Cross-Validation

The cross-validation process is utilized to assess the conduct of the scheme and the traditional custom is the random-split cross-validation. But the random-split cross-validation approach is usually too buoyant for the evaluation of model predictive outcome as it undermines the covariate alterations in drug development via combining unrelated series’ data [211]. On the other hand, the paradigm of the time-split cross-validation was put forward where the datasets were apportioned into training and test groups depending upon the experimental time order of the data [212]. Sheridan et al. compared varied cross-validation algorithms employed to assess the conduct of the QSAR model and their outcomes indicated that the *R*2 value obtained by time-split cross validation scheme was more representative of the actual prospective predictive value [213]. Steered by this outcome, Ma et al., engaged the time-split cross-validation instead of traditional random-spilt cross-validation to appraise the conduct of the deep neural network (DNN) in mimicking the pragmatic hit-to-lead schema [8]. For all of these studies, experimental time is a crucial vital attribute, and time-split cross validation must be executed in drug discovery when the data of experimental time information is at one’s disposal.

### 3.4. What It Takes to Train the Deep Neural Networks

Despite the fact that DL models outclass various conventional ML algorithms, they still invoke many more parameters and unrelated architectures, which leads to several problems during training, specifically in the situations when the samples are inadequate or the feature matrix is meagre. The training process might only obtain a ‘local optimum’ and the accurateness is inadequately valid. For combating this issue, the unsupervised pre-training approach like “deep belief network” has been recommended to upgrade the parameter booting, and the outcomes hint that the approach has been extra efficacious in comparison with the random initial values [153]. A study hinted that the dropout blueprint could efficiently avert overfitting during the QSAR dataset training [8]. Furthermore, in comparison with the “sigmoid action function”, “Rectified Linear Unit (ReLU)” action function has added relevance in the context of the QSAR assignments due to its benefits in forestalling the ‘gradient disappear’ as well as ‘local optimum’.

### 3.5. The Accessible Drug Design AI Source Code

In the pharmaceutical sector, the business advantages of computer software driven drug design is proven. But a great many software originators are motivated to disseminate their programs on GitHub or various open-source repositories, to combine the AI algorithms with drug design approaches. Many open-source execution platforms for AI-facilitated drug design paradigms have been outlined in Table 1. Such open-source repositories will boost the pervasive operationalization of AI technologies in this arena.

## 4. Contribution of AI in the Lifecycle of Pharmaceutical Items

This section outlines AI solutions in pharmaceutical products’ lifecycle that could find numerous implementations, but none of the available market solutions cover them all. Natural Language Processing (NLP) allows document summarization, document generation, and Named Entity Recognition (NER) based on novel Bidirectional Encoder Representations from Transformers (BERT) and Generative Pre-trained Transformer (GPT). It can be used for Real World Evidence (RWE)-based trials, reports, and summaries generation. Random Forest (RF), Naive Bayes (NB), and Support Vector Machine (SVM), as well as other methods, could be used for a large amount of unstructured information analysis for new drug target identification. Deep Neural Networks (DNNs), Reinforcement Learning (RL), and Principal Component Analysis (PCA) are most useful for novel molecule generation in silico and their activity prediction. Drug repositioning and repurposing could be done with text mining, coupled with Feed-forward Neural Network (FNN). Generation of synthetic biology is based mostly on NLP implementations for RNA-based sequencing. Clinical trials utilize Real World Data (RWD) and RWE approaches with AI, NLP, and NER support. Image classification with Convolutional Neural Networks (CNNs) can automatically discover, generate, and learn features of images which are useful in pre-clinical and clinical trial results processing. Personalized therapy could be aided with Neural Network (NN) patient risk prediction and multiple factors analysis, including genetics. Drug dispensing control is based on Electronic Medical Record (EMR) analysis for counterindications and drug combination interactions. Additionally, ML and AI technologies could be used for monitoring and predicting epidemic outbreaks around the world to align pharmaceutical development.

### 4.1. AI in Promoting Pharmaceutical Product Advancement

The identification of a novel drug compound depends upon its consequent embodiment in a proper dosage formulation with preferred delivery attributes. From this aspect, AI can oust the earlier trial and error method [214]. Many computational techniques can iron out issues experienced in the formulation design aspect, like stability problems, porosity, dissolution, etc., utilizing Quantitative Structure Property Relationship (QSPR) [215]. Decision-support systems invoke rule-grounded algorithms to decide the class, attributes, and amount of the excipients banking upon the physicochemical features of the medication and act via a feedback system to supervise the whole mechanism and periodically adjust it [216].

An amalgamation of “Model Expert Systems (MES)” with ANN to engender a mixed approach for the advancement of direct-stuffing of hard gelatin piroxicam capsules in conformity with the stipulations of its dissolution parameters has been reported. The MES determines options and propositions for formulation advancement depending upon the input feed criteria. On the contrary, ANN employs backpropagation training to connect formulation criteria to the preferred feedback, managed with the control module in tandem, to assure convenient formulation advancement [214].

Multiple mathematical means, such as Computational Fluid Dynamics (CFD), Discrete Element Modeling (DEM), and the Finite Element Method (FEM) have been employed to probe the effect of the flow characteristics of the powder upon the die-stuffing and method of tablet compression [217,218]. In addition, CFD could be employed to examine the influence of tablet shape/size upon the dissolution parameters [219]. The amalgamation of such mathematical paradigms with AI may turn out to be of great benefit for the swift manufacture of pharmaceutical items.

### 4.2. Contribution of AI towards Manufacturing of Pharmaceutical Products

In view of the rising intricacies of production systems coupled with an incremental need for optimization and improved product standards, contemporary production approaches are attempting to transfer human know-how to machines, frequently transforming the production aspects [220]. The integration of AI in manufacturing systems can hold a plethora of advantages for the pharmaceutical sector. Aids like CFD engage “Reynolds-Averaged Navier-Stokes” solvers technique which probes the effect of agitation and stress grades in various equipage (like stirred tanks), harnessing the mechanization of a multitude of pharmaceutical processes. Identical processes, like ‘direct numerical simulations’ as well as ‘large eddy simulations’, employ cutting-edge schemes to iron out convoluted flow issues in production [217].

The innovative“Chemputer”system facilitates digital mechanization in the synthesis and production of molecules, unifying many chemical signatures and executing by utilizing a scripting software code named “Chemical Assembly (ChASM)” [221]. This has been utilized opportunely for the formation and production of diphenhydramine hydrochloride, rufinamide, and sildenafil, with the harvest and cleanness notably identical to hand-operated synthesis [151]. The predicted achievement of granulation in granulators of volumes varying between 25–600 L could be accomplished effectively by AI technical knowledge [222]. The technical knowledge and neuro-fuzzy logic links vital parameters to their output. This system formulated a polynomial relationship for the prognostication of the ratio of the granulation fluid to be poured, desired speed, as well as the impeller diameter parameters in both geometrically identical and non-identical granulators [223].

“Discrete Element Modeling (DEM)”is extensively employed in the pharmaceutical sector, such as in evaluating the partition of powders constituting a binary mixture, the fallout of altering blade speed and geometry, foretelling the probable route of the tablets for the encapsulation procedure, together with scrutiny of time expended by tablets in the spray section [217]. ANNs, coupled with fuzzy paradigms, examined the interrelationship among machine settings as well as the issue of capping to pare tablet capping on the production line [224].

AI capabilities such as meta-classifier and tablet-classifier could facilitate the management of the quality benchmark of the ultimate output, like pointing to a probable aberration in tablet production [225]. A patent has been applied for, establishing a process skillful in identifying the most exclusive amalgamation of drug and dosage schedule for individual patients, employing a processor culling patient data, and configures the preferred transdermal patch as required [226].

### 4.3. Role of AI in Managing and Ensuring Quality

Production of the preferred item from the raw goods requires a harmonization of multiple criteria [225]. Stringent quality checks on the items, as well as upkeep of batch-to-batch constancy, behooves hand-operated intervention. This might not be the ideal method in each instance, signifying the necessity for AI engagement during this time [217]. The FDA updated the “Current Good Manufacturing Practices (cGMP)” by suggesting a ‘Quality by Design’ scheme to comprehend the pivotal activity and explicit standards that regulate the ultimate nature of the pharmaceutical product [227]. A blend of human endeavor and AI have been utilized, wherein first-round data from manufacturing sets were scrutinized and decision trees originated. They were subsequently transliterated into axioms and explored by the operators to facilitate the manufacturing cycle afterwards [225]. A scientific document reviewed the dissolution characteristics, a barometer of batch-to-batch constancy of theophylline pellets with the help of ANN, that accurately presaged the dissolution of the examined formulation, the inaccuracy being< 8% [228].

AI can also be executed for the governance of in-line production schemes to accomplish the preferred product quality [227]. ANN-facilitated surveillance of the freeze-dehydrating approach is utilized, which implements a merger of self-adaptive evolvement together with local search as well as backpropagation algorithms. Such methods can be employed to foretell the temperature and desiccated-cake thickness at a later time point (t +Dt) for a specified group of operating characteristics, ultimately facilitating in imposing a vigil on the eventual product standards [229].

An automatic data entry algorithm, like an “Electronic Lab Notebook”, in concert with refined, resourceful mechanisms, can secure the quality guarantee of the produce [230]. In addition, data mining and multiple knowledge discovery methodologies in the “Total Quality Management (TQM)” expert process may be utilized as worthwhile avenues in arriving at convoluted judgments, crafting advanced technologies for astute quality management [231].

### 4.4. Role of AI Algorithms in Determining Clinical Trial Blueprints

Clinical trials are aimed at demonstrating the safety and efficacy of a medication in humans for a specific ailment and need 6–7 years together with a significant financial outlay. But only 10% molecules tested in such trials achieve fruitful approval, which is a gigantic failure for the industry [232]. These losses can arise due to incorrect patient choice, paucity of technical infrastructure, and poor facilities. But, with the colossal digital medical information accessible, these setbacks may be curtailed by utilizing AI [233].

The recruitment of patients consumes about 33% of the clinical trial duration. The fruition of a clinical trial may be facilitated by appropriate patient enrollment, which contrarily results in ~86% of non-fruition scenarios [234]. AI may help in choosing only a selective diseased populace for Phase II and III clinical trial enrollment by utilizing patient-pertinent genome-exposome feature scrutiny, which could facilitate advanced augury of the existing drug targets in the subjects chosen [59,233]. Preclinical scrutiny of molecules and also identifying lead compounds prior to the initiation of clinical trials by employing adjunct attributes of AI, like predictive ML and alternative inferencing algorithms, aid in the advanced forecasting of lead molecules which would make the cut in clinical trials in the chosen patient population [233].

Drop out of patients in clinical trials contributes to the non-fruition of about one-third of clinical trials, resulting in auxiliary enrolment needs for the culmination of the trial, with consequent improvidence of time and finances. Such issues can be obviated by tight surveillance of the patients and facilitating them in complying with the rightful protocol of the clinical trial [234]. Mobile software has been introduced by “AiCure” which checked usual medication use by schizophrenia patients in a Phase II trial, with consequent augmentation of the compliance frequency of patients by 25%, assuring a fruitful conclusion of the clinical trial [59].

### 4.5. Role of AI in Pharmaceutical Product Management

#### 4.5.1. Role of AI in Market Positioning

Market alignment is the scheme of engendering a uniqueness of the marketed product to entice buyers to purchase it, making it a mandatory component in most business tactics for organizations to build their own novel niche [235,236]. This strategy was utilized for marketing of prime brand Viagra, in which the marketing firm targeted it not only for addressing men’s erectile impairment, but also for adjunctive issues influencing quality of life [237].

Utilizing technology coupled with e-commerce as a launchpad, it has become smoother for organizations to acquire an instinctive acclaim of their brand identity in the public sphere. Firms harness search engines among many available technological pulpits to take up an eminent place in online marketing and aid in the market alignment of the product, as also established by the “Internet Advertising Bureau”. Firms repeatedly endeavor to classify their websites better than those of competitor firms, providing identity to their brand in an abbreviated timeline [238].

Other approaches, like statistical assessment techniques, particle swarm optimization schemes (documented in 1995 by Eberhart and Kennedy) together with NNs, gave a superior opinion about markets. Such approaches can aid in selecting the marketing blueprint for the product attuned to precise consumer-demand prognostication [239].

#### 4.5.2. Role of AI in Market Forecasting and Scrutiny

The prosperity of a firm rests upon the ongoing advancement and augmentation of its commercial interests. Even with outlay of massive funds, R&D harvest in the pharmaceutical sector is declining owing to the inability of firms to adapt to current marketing methodologies [240]. The evolution of digital technologies, named the “Fourth Industrial Revolution”, is facilitating novel digitalized marketing through a multimodal decision-making scheme, which obtains and evaluates statistical and mathematical data and executes human interpretations to enable AI-enabled decision-making paradigms hunt for fresh marketing prospects [241].

AI also facilitates a detailed scrutiny of the core needs of a product from a customer’s viewpoint and also in comprehending the requirement of the market, which helps in decision-making utilizing prediction models. This process is also capable of foretelling sales and evaluating the market. Software engaging AI employ consumers and engender knowledge among healthcare professionals by exhibiting commercials targeting them to the product section with one click [242]. Moreover, these approaches engage natural language-processing (NLP) algorithms to scrutinize keywords fed by buyers and link these to the possibility of buying the product [243,244].

Many businesses to business (B2B) firms have declared self-use platforms that permit free survey of health products, readily located by providing its specification, accept orders, as well as monitor their transportation logistics. Pharmaceutical organizations are also putting forward their online sites like “1 mg”, “Medline”, “Netmeds”, and “Ask Apollo”, to address the unfulfilled patient requirements [241]. Prognostication of the selling space is also imperative for many pharmaceutical trading firms, with the capability to execute AI in the field, in the manner of “Business intelligent Smart Sales Prediction Analysis”, which utilizes a merger of time series prediction and real-time utilization. This assists pharmaceutical firms to foretell the trade of products aforetime to forestall expenses of surplus buildup or avert buyer disadvantage due to shortfall [245].

#### 4.5.3. Role of AI in Product Cost

Depending upon the market assessment and cost acquired in the advancement of the pharmaceutical goods, the organization decides the ultimate cost of the item. The crucial notion in implementing AI to resolve this cost is utilizing its prowess to simulate the cognition of a human specialist to evaluate the criteria that govern the valuation of a product following its production [245]. Issues, like financial outlay in the course of research and advancement of the medication, rigorous price control plans in the relevant country, period of the exclusivity duration, market stake of the improvised agent after a year prior to patent expiration, costing of the reference item, and price-determining statutes control the cost of branded as well as generic medications [246].

In ML, massive bodies of statistical data, like product advancement expenditure, product need in the market, itemization record expenses, manufacturing expenses, and competitors’ product cost, are evaluated using the algorithm, consequently evolving software for predicting the product cost in the aftermath. AI algorithms such as “In competitor”, floated by “Intelligence Node” (set up in 2012), is a total market competitive savvy package that scrutinizes the competitor costing information and aids market players and brands to govern the competition. “Wise Athena” and “Navetti PricePoint” facilitate the user to set the costing of their item, implying that pharmaceutical establishments can embrace the same to aid product pricing [247].

### 4.6. A Snapshot of AI-Based Advanced Implementations

#### 4.6.1. Drug Delivery Technologies Engaging AI-Grounded Nanorobots

Nanorobots are composed of primarily integrated circuits, power source, sensors, as well as a protected auxiliary data alternative, which are abetted by computational know-how, like AI [248,249]. Such algorithms are trained to avert the encounter, target determination, identification and association, and ultimately purging out from the body. Advancements in nano/microrobots endow such contraptions with the capability to cruise to the focused locus depending upon physiological circumstances, like pH, hence bettering the efficacy and curbing adverse actions on body systems [249]. Evolution of body-fixable nanorobots initiated for controlled distribution of medicaments and genes behooves review of criteria like dose tailoring, continued drug transmission, and modulated release, as well as the discharge of the drugs needing mechanization managed by AI algorithms, like NNs, integrators, and fuzzy logic [250]. Body-fixable microchips are employed for programmed drug transmission and to identify the position of the implant within the body.

#### 4.6.2. Role of AI in Concerted Drug Delivery and Augury of Synergism/Antagonism

Several drug combinations have been authorized and offered for sale to counter complex afflictions, like TB and cancer, since they are capable of furnishing a synergistic action for swift improvement [251,252]. The choice of appropriate and promising medications for combination needs high-throughput scrutiny of a sizable quantum of medications, leading to a labor-intensive mechanism; for instance, cancer treatment needs six or seven medicinal agents for combination chemotherapy. ANNs, network-dependent modeling, and logistic regression could enable screening drug combos and upgrade general dose schedules [251,253]. Rashid et al., proposed a ‘quadratic phenotype optimization scheme (QPOS)’which identifies efficacious combination treatment for the management of bortezomib-resistant multiple myeloma utilizing a selection of 114 FDA-authorized agents. This paradigm endorsed the pairing of mitomycin C (MitoC), with decitabine (Dec) as the leading two-agent combo and MitoC, mechlorethamine, with Dec as the preferred three-agent combo [252].

Drug administration in combination may be more effective if assisted by information on the synergism or antagonism of drugs transmitted concomitantly. The “Regulator Inference Algorithm utilizes ‘Master regulator genes’ to competently foretell 56% of synergistic action. Alternative approaches, like Network-based Laplacian regularized Least Square Synergistic (NLLSS) drug combination, as well as ‘random forest (RF)’, may also be utilized for the purpose [253].

Li et al. advanced a synergistic drug assortment paradigm utilizing RF for the augury of synergistic anticancer drug combos. This exemplar was engendered grounded upon gene expression attributes and many networks, so that the researchers could effectively foretell 28 synergistic anticancer combos. They have documented three such assortments, even if the rest could also ultimately turn out to be critical [66]. Furthermore, an ML implementation scheme, termed the Combination Synergy Estimation, is capable of presaging promising synergistic antimalarial drug assortments from a library group of 1540 antimalarial drug molecules [254].

#### 4.6.3. The Materialization of AI in Nanomedicine

Nanomedicines utilize nanotechnology and medications for the diagnosis, treatment, and surveillance of convoluted afflictions, like malaria, cancer, HIV, many inflammatory maladies, and asthma. Of late, nanoparticle-modulated drug delivery has assumed dominance in the arena of therapeutics and diagnostics as they have improved therapeutic effectiveness [252,255]. A merger of nanotechnology with AI may afford answers to various issues in formulation advancement [256].

A nanosuspension of methotrexate has been algorithmically methodized by examining the energy emanating from the admixing of the drug molecules, examining the factors that could favor the formulation clumping [215]. ‘Coarse-grained simulation’, in concert with chemical estimation, can assist the interactive evaluation of drug-dendrimer and appraisal of drug encapsulation inside the dendrimer. Furthermore, software such as LAMMPS and GROMACS4 could be utilized to probe the punch of surface chemistry on the cellular uptake of nanoparticles [215].

AI enabled the formation of silicasomes, which is a blend of internalizing arginine-glycine-aspartic acid sequences (iRGD), a tumor-penetrating peptide, and multifunctional mesoporous silica nanoparticles charged with irinotecan. This internalization of silicasomes may be enhanced three- or four-fold as iRGD promotes silicasometranscytosis, with enhanced treatment results and favorable long-term survival [255].

## 5. The Market Potential of AI Applications for Drug Discovery and Development

To curtail the fiscal expenses and possibility of losses which are associated with Virtual Screening (VS), pharmaceutical enterprises are switching over to AI applications. The AI market witnessed an upsurge from USD 200–700 million between 2015–2018, and this is anticipated to rise by 2024 to USD 5 billion [257]. A 40% estimated surge from 2017–2024 implies that AI will possibly refashion the medical and pharmaceutical arenas. Many pharmaceutical firms have devoted or/and are maintaining financial commitment in AI and in addition cooperated with AI providers for engendering indispensable healthcare paraphernalia. Cooperation between DeepMind Technologies, a branch of Google, and the Royal Free London NHS Foundation Trust for the abatement of acute kidney injury, has been an exemplary instance. Primary pharmaceutical firms and AI vendors have been specified in Table 2 [258].

## 6. Continuing Bottlenecks in Accepting AI: Hints on Methods to Conquer

In spite of rapid advancements in AI and ML algorithm technologies implemented in the pharmaceutical industry, there persist numerous threats regarding the implementation and assimilation of these technologies into the drug discovery process specifically and the pharmaceutical industry in general.

One problem is sloppy data integration. This issue arises from diversity existing between datasets, which may constitute raw data, processed data, metadata, or candidate data. Such datasets should be accumulated and collated for effective analysis, but presently, there exists no validated method of doing so. This is imperative prior to initiation of the drug discovery process, as without appropriately formatted data, the output of the ML algorithms will be imprecise. More efficient methods for integrating available data into data banks before the drug discovery process is initiated are therefore necessary.

A separate recognized issue is occupational and skillset immobility: many people presently engaged in the pharmaceutical sector lack the mandatory skillsets or the qualifications required for operating AI systems. A good number of the workforce are proficient in data science, while others in molecular chemistry and biology, though few are experts in both domains, with the optimum amalgamation of skills to engage AI from a pharmaceutical context. An awareness of the underlying chemistry is imperative for the origination of relevant algorithms, and vice versa.

Each firm applies their own proprietary AI algorithms which are unavailable in the public domain. Thus, there is skepticism about ML and AI in the pharmaceutical industry stemming out from a deficient comprehension about the methodology of algorithms, termed as the “black box” phenomenon, and agnosticism for the results generated. Those who are skeptical may be hesitant to engage the data originating from AI and ML, squandering both time and money, and impeding the forward progression of the industry with regards to efficiency.

The absolute triumph of AI banks upon the accessibility of a massive volume of data as such data are utilized during consequent training subjected to the algorithm. Availability of data from numerous database vendors can inflict additive costs to a firm, and the data must also be dependable and excellent quality to assure precise outcome forecasting. Further bottlenecks that hinder full-blown acceptability of AI in the pharmaceutical sector comprise the dearth of trained manpower to implement AI-based systems, restricted financial resource base for small establishments, worries of substituting humans with subsequent job losses, lack of confidence in the data churned out by AI, as well as the “black box effect” (i.e., the mechanisms contributing to the compiled outcomes which are generated as a result of the AI algorithm) [18].

Mechanization of many steps in drug advancement, production, and supply networks, clinical trials, and trading will occur over time, but all such activities get incorporated in the umbrella of ‘narrow AI’; where AI has to be schooled utilizing a massive amount of data and, hence, makes it appropriate for a specific assignment. Hence, human mediation is compulsory for the effective application, advancement, and execution of the AI algorithm. But the apprehension of retrenchment could be a delusion considering that AI is recently assuming iterative tasks, while sparing liberty for human intellect to be utilized for advancing more convoluted judgements and ingenuity.

Notwithstanding, AI has been accepted by numerous pharmaceutical organizations, and it is anticipated that earnings of about USD 2.2 billion will be realized by 2022 via AI-grounded fixes in the pharmaceutical arena, with a financing in excess of USD 7.20 billion embracing 300+ pacts during 2013–2018 by the pharmaceutical business [259]. Pharmaceutical businesses require transparency regarding the promise of AI algorithms in innovating troubleshooting fixes to complications once it has been applied, in concert with comprehending the justifiable standards that can be accomplished. Talented data scientists, software engineers equipped with a solid understanding of AI system tools, and a transparent comprehension of the objectives and R&D focus of business models will enable the advances and engagement that the AI platform promises.

## 7. Conclusions and Future Promise

The progress of AI, together with its impressive tools, regularly aiming to curtail bottlenecks encountered by pharmaceutical organizations, affecting the drug advancement pipelines in concert with the long-term lifecycle of the merchandise, may justify the spurt in the quantum of start-ups in this arena [260]. The present healthcare arena is experiencing numerous contorted threats, like the rising prices of medications and treatments, and society requires definitive, noteworthy action in such fields. Consequent upon the incorporation of AI in the production of pharmaceutical goods, personalized medications with the apt dose, release attributes, and varied needed facets may be produced in accordance with individual patient demand [234]. Utilizing the current AI-aided algorithms should abbreviate the duration necessary for the goods to reach the market, as well as also enhance the quality of goods and the comprehensive security of the manufacturing scheme, and lead to augmented usage of accessible resources together with being cost-efficient through underscoring and advancing the criticality of mechanization [261].

The most serious apprehension concerning the inclusion of these platforms is the job cuts that are anticipated to emerge and the rigorous practices mandated towards application of AI. But, these technologies are proposed in order to render the task effortless and not to totally oust humans [262]. Apart from facilitating swift and issue-free hit compound determination, AI may also furnish recommendations of synthesis pathways of these agents together with the augury of the preferred chemical architecture and a comprehension of drug-target associations and the pertinent SAR.

AI is also capable of proposing dominant inputs to the subsequent inclusion of the originated drug in its pertinent dosage form and its refinement, in concert with facilitating swift decision-making, culminating in rapid output of enhanced-quality goods together with promise of batch-to-batch dependability. AI may in addition add to instituting the safety and effectiveness of the agents in clinical trials, coupled with assuring optimum alignment and pricing in the market via extensive market scrutiny and forecasting. Regardless of the truth that there are no drugs presently on the market originated with AI-enabled schemes, distinct challenges prevail with regard to the application of this technology, it is possible that AI will mature into a precious tool in the pharmaceutical sector in the imminent future.

## Figures and Tables

**Table 1:** Enumeration of AI-Aided Computational Tools for Facilitating Drug Discovery.

Tools	Feature(s)	Website(s)	Reference(s)
AlphaFold	Protein 3D (tertiary) structure presage employing DNN	https://deepmind.com/blog/alphafold (accessed on 28 November 2022) https://www.sciencemag.org/news/2018/12/google-s-deepmind-aces-protein-folding (accessed on 28 November 2022)	[151]
Chemputer	An exhaustive regulated schema for documenting a chemical synthesis method (Furnishes comprehensive compound synthesis recipe)	https://zenodo.org/record/1481731 (accessed on 28 November 2022)	[149]
Conv_qsar_fast	Foretells molecular attributes aided by CNN algorithm	https://github.com/connorcoley/conv_qsar_fast (accessed on 28 November 2022)	[130]
Chemical VAE	Mechanized chemical crafting employing variational autoencoder (VAE)	https://github.com/aspuru-guzik-group/chemical_vae (accessed on 28 November 2022)	[133]
DeepChem	A Python-aided AI technique for various drug discovery workflow predictions utilizing a DL algorithm for molecule recognition	https://github.com/deepchem/deepchem (accessed on 28 November 2022)	[152]
DeepNeuralNet-QSAR	Foretells molecular activity engaging multilevel DNN	https://github.com/Merck/DeepNeuralNet-QSAR (accessed on 28 November 2022)	[153]
DeepTox	Toxicity predictions of chemical agents utilizing a DL algorithm	www.bioinf.jku.at/research/DeepTox (accessed on 28 November 2022)	[154]
DeltaVina	Presages small molecule interaction affinity with drug employing an amalgamation of random forest (RF) as well as AutoDock scoring function)	https://github.com/chengwang88/deltavina (accessed on 28 November 2022)	[111]
Hit Dexter	ML schemes for the presage of compounds that could be sensitive to biochemical assays by engaging ML techniques	http://hitdexter2.zbh.uni-hamburg.de (accessed on 28 November 2022)	[155]
InnerOuterRNN	Foretells the chemical, physical, and biological attributes utilizing inner- and outer RNNs	https://github.com/Chemoinformatics/InnerOuterRNN (accessed on 28 November 2022)	[156]
JunctionTree VAE	De novo molecule origination utilizing junction tree variational autoencoder (VAE)	https://github.com/wengong-jin/icml18-jtnn (accessed on 28 November 2022)	[157]
Neural Graph Fingerprints	Attribute augury of novel molecules employing CNN algorithms	https://github.com/HIPS/neural-fingerprint (accessed on 28 November 2022)	[158]
NNScore	Foretells the affinity of protein–ligand binding utilizing neural network-aided scoring function	http://rocce-vm0.ucsd.edu/data/sw/hosted/nnscore/ (accessed on 28 November 2022) http://www.nbcr.net/software/nnscore (accessed on 28 November 2022)	[159]
Open Drug Discovery Toolkit (ODDT)	An exhaustive toolkit utilized for chemoinformatics and molecular modelling employing random forest score (RF)-Score as well as NNScore	https://github.com/oddt/oddt (accessed on 28 November 2022)	[160]
ORGANIC	A competent molecular generation tool to originate molecules with favourable attributes employing ML schemes	https://github.com/aspuru-guzik-group/ORGANIC (accessed on 28 November 2022)	[161]
PotentialNet	Foretells ligand-binding affinity engaging graph CNN	https://pubs.acs.org/doi/full/10.1021/acscentsci.8b00507 (accessed on 28 November 2022)	[162]
PPB2	Poly-pharmacology prediction employing nearest neighbour as well as ML schemes	http://ppb2.gdb.tools/ (accessed on 28 November 2022)	[163]
QML	A Python toolkit for quantum ML (utilizing qubits leading to incremented computational speed, data storage capacity, and learning optimization)	https://www.qmlcode.org (accessed on 28 November 2022) https://github.com/qmlcode/qm (accessed on 28 November 2022)	[164]
REINVENT	De novo design of molecule employing RNN (recurrent neural network) as well as RL (reinforcement learning)	https://github.com/MarcusOlivecrona/REINVENT (accessed on 28 November 2022)	[165]
SCScore	A scoring scheme to figure out the synthesis complexity of a compound	https://github.com/connorcoley/scscore (accessed on 28 November 2022)	[166]
SIEVE-Score	An upgraded technique of structure-aided virtual screening through interaction-energy-based learning	https://github.com/sekijima-lab/SIEVE-Score (accessed on 28 November 2022)	[167]

**Table 2:** Partnerships of AI establishments with pharmaceutical firms.

Company/ Firm	Utilization of AI	Partnership with the Pharmaceutical Establishment	Platform Advanced/Lead Agents for Clinical Trials
Numerate San Francisco, CA 94107, USA	A scheme for AI-facilitated drug design addressing oncology and gastroenterology specialities	Takeda	Agent S48168 in Phase 1 of clinical testing for Ryanodine receptor 2
Numerate San Francisco, CA 94107, USA	A scheme for AI-facilitated drug design addressing oncology and gastroenterology specialities	Servier	Drug advancement related to oncology, central nervous system, and gastroenterologic maladies
Atomwise San Francisco, CA 94103, USA	A scheme for AI-enabled structural modelling	Lilly	Agent BBT-401 in Phase 2 of clinical testing
Atomwise San Francisco, CA 94103, USA	A scheme for AI-enabled structural modelling	Bridge Biotherapeutics	Augmentation of Pellino Inhibitor Pipeline; Agent BBT-401 evaluated in Phase-2a of clinical testing
Benevolent AI London, UK	AI-facilitated Judgement Augmented Cognition System (JACS) for originating and advancing novel clinical lead agents effective in neurodegenerative ailments	Janssen	Fresh set of drug compounds to be advanced via such collaboration
Benevolent AI London, UK	AI aided schemes to advance novel clinical lead agents effective in chronic kidney ailments	AstraZeneca	Drug candidate evaluated in Phase 2b clinical testing as a lead agent effective in chronic kidney ailments
Exscientia Oxford, UK	A scheme for AI-enabled drug discovery and lead refinement	Sanofi	Drug Discovery Research in obsessive-compulsive disorder, Agent DSP-1181 in Phase I clinical testing. Advance Centaur Chemist™ scheme for AI-enabled drug discovery
IBM Watson Health Cambridge, MA 02142, USA	Furnishes a scheme for clinical and health-associated data evaluation	Pfizer	Accelerating drug discovery efforts in immuno-oncology
IBM Watson Health Cambridge, MA 02142, USA	Furnishes a scheme for clinical and health-associated data evaluation	Novartis	Real-time surveillance of patients to augment breast cancer patient intervention results
Microsoft Redmond, WA 98052, USA	A scheme for image processing as well as cell and gene-aided therapeutic interventions	Novartis	Engendering an AI Innovation lab to augment the drug discovery mechanism as well as its commercialization
Owkin Broadway, New York, NY, USA	Furnish a scheme for clinical testing aided by ML technique	Roche	Originated and advanced Owkin’s Studio platform utilizing AI technology
Sensyne health Headington, Oxfordshire, UK	A tool serving clinical AI schemes	Bayer	Originated and advanced Sensyne Health’s proprietary clinical AI technology package
XtalPi Shenzhen, Guangdong, China	A package enabling Target identification and validation incorporating QM as well as ML schemes	Pfizer	Presage and refinement of crystalline entities of drug candidates utilizable in early stages of drug screening
BioXcel therapeutics New Haven, CT, USA	A scheme facilitating drug discovery services incorporating AI mechanisms	Pfizer	Lead agent BXCL501-in assessment in Phase 3 clinical testing; Drug agent BXCL701-in assessment in Phase 2 clinical assessment

## Data Availability

Not applicable.
